# Patient Communication Outside of Visits: Implications for Remote Monitoring and Digital Care Models in Myasthenia Gravis

**DOI:** 10.1002/mus.70341

**Published:** 2026-07-16

**Authors:** Maike Stein, Lea Gerischer, Yvonne J. M. Campman, Anna Rostedt Punga, Pushpa Narayanaswami, Andreas Meisel, Martijn R. Tannemaat, Sophie Lehnerer

**Affiliations:** ^1^ Charité—Universitätsmedizin Berlin, Corporate Member of Freie Universität Berlin and Humboldt‐Universität Zu Berlin, Department of Neurology With Experimental Neurology Berlin Germany; ^2^ Charité‐Universitätsmedizin Berlin, Corporate Member of Freie Universität Berlin and Humboldt‐Universität zu Berlin, Neuroscience Clinical Research Center Berlin Germany; ^3^ Department of Neurology Leiden University Medical Center Leiden the Netherlands; ^4^ Department of Medical Sciences Clinical Neurophysiology, Uppsala University Uppsala Sweden; ^5^ Department of Neurology Beth Israel Deaconess Medical Center/Harvard Medical, School Boston Massachusetts USA; ^6^ Center for Stroke Research Berlin, Charité—Universitätsmedizin Berlin Berlin Germany; ^7^ Berlin Institute of Health at Charité—Universitätsmedizin Berlin, Digital Health Center Berlin Germany

**Keywords:** monitoring, myasthenia gravis, platform, telemedicine, wearables

## Abstract

**Introduction/Aims:**

Myasthenia gravis (MG) is a rare autoimmune disorder requiring individualized, specialized treatment, yet access to specialized care is limited and documentation fragmented. Several remote monitoring tools have emerged to address this gap, yet their integration into clinical care remains challenging. The aim was to better understand the nature and context of care‐related interactions in order to inform the development of clinically meaningful and patient‐centered digital health solutions for MG.

**Methods:**

We conducted a survey among physicians and nurses at a tertiary MG center over an eight‐week period regarding care‐related inquiries, documented using a structured report form that captured the topic, urgency, communication channel, processing time, and clinician involved.

**Results:**

About 576 care‐related inquiries were recorded. Most inquiries addressed organizational (55.4%) or medical (41.3%) issues. Among medical topics, symptom worsening (30.7%) and treatment‐specific questions regarding FcRn and complement inhibitor therapy (19.3%) were most frequent. Organizational topics were most frequently about appointments (43.6%) and prescriptions (35.7%). A total of 115 inquiries (20.0%) were time‐critical (requiring same weekday response). Physicians answered 74.3% of inquiries. The most common communication channels were e‐mail (42.4%) and telephone (29.0%), followed by the hospital information system (13.7%) and a telemedicine platform (6.9%). Medical inquiries took twice as long to process/respond as organizational ones (median 10 vs. 5 min).

**Discussion:**

Our findings show that care‐related inquiries in MG are diverse and answered through various communication channels. Remote monitoring solutions hold promise to complement existing care structures in MG and could support timely and structured care but require human‐guided processes.

## 1 Introduction

Patients with myasthenia gravis (MG) require specialized and individual care delivered in a timely and patient‐centered manner. Yet, many patients face limited access to specialized care, due to geographic or infrastructural barriers, and there is a lack of continuous disease monitoring between clinic visits [1]. Studies have highlighted a substantial disease burden in MG [2] and a high demand for personalized counseling across various topics [3]. However, symptom monitoring is usually still restricted to brief, infrequent clinic visits that may not coincide with periods of clinical instability or patient‐perceived need. In response to these challenges, various telemedical and remote monitoring approaches have been proposed to improve care in MG [4]. These include structured remote assessments and patient‐performed measures to capture disease activity [5, 6, 7], as well as app‐ and smartphone‐based tools enabling patients to document symptoms in home settings [8, 9, 10]. Comprehensive telemedicine platforms tailored to MG, combining secure patient‐physician communication, collection and patient‐reported outcome measures (PROMs) and sensor‐based data from wearables are also being piloted in specialized centers [11, 12]. While promising tools have been evaluated in feasibility studies, there is a lack of data on how patients engage with healthcare teams outside scheduled visits. To address this gap, we conducted a survey assessing all care‐related inquiries to a tertiary MG center in Germany over an eight‐week period. The aim was to better understand the nature and context of these care‐related interactions in order to inform the development of clinically meaningful and patient‐centered digital health solutions for MG.

## 2 Methods

### 2.1 Design

We conducted an observational survey over an eight‐week period (March 1, 2025 to April 30, 2025) at a tertiary MG center at Charité—Universitätsmedizin Berlin, Germany. This center provides care for approximately 1000 outpatients and 250 inpatients with MG per year. All care‐related inquiries, including their processing and response by the MG team (nurses, physicians) across various communication channels were documented and analyzed qualitatively. This also included inquiries via MyaLink, a telemedicine platform developed by the study team at Charité tailored for MG patients, which enables the structured collection of patient‐reported outcomes and clinical parameters, and also incorporates a communication tool facilitating direct patient–healthcare provider interaction with the MG center. All incoming inquiries through the various communication channels were usually answered by the MG team member who received the patient's message. Clearly organizational matters (e.g., appointment coordination and prescriptions) were mainly managed by nurses. Inquiries involving medical decision‐making were handled by physicians. If nurses received inquiries requiring medical input, they consulted the responsible physician before responding, thereby ensuring medical oversight for clinically relevant concerns.

According to local regulations, formal ethics committee approval was not required, as the study assessed internal workflow processes and healthcare professional responses to patient inquiries without involving patient‐identifiable data, further direct patient contact, or any intervention.

### 2.2 Data Collection

Data entry was performed by six MG‐trained specialists (physicians) and three study nurses, using a structured evaluation form (Table S1). The structured evaluation form documented the communication channel used for responding to each inquiry (completed by the healthcare professional who handled the request), not the original channel through which the inquiry was initiated. “Time‐critical” requests were pre‐defined before study initiation as inquiries requiring a response within the same working day, with urgency assessed by the clinician (nurse or physician) who first reviewed the request.

### 2.3 Data Analysis

Descriptive statistics were used to characterize request types and communication patterns (SPSS version 29.0.0. [IBM, Armonk, NY]).

## 3 Results

About 576 care‐related inquiries were recorded (Table 1). A total of 64 participant‐weeks were included (summing across all participants and counting only weeks with a complete dataset provided by a physician or nurse), with data missing for eight of those weeks (four due to vacation leave and four due to excessive workload precluding questionnaire completion). Physicians documented between 3.9 and 18.3 (min, max) inquiries per week, while study nurses recorded between 3.8 and 10.9 (min, max) weekly inquiries. Inquiries most frequently concerned organizational and medical issues (Table 2). Among medical inquiries, the most frequently addressed topics included MG symptom worsening and treatment‐specific questions regarding FcRn or complement inhibitor therapy (e.g., inquiries related to perceived loss of treatment effect, side effects, or general therapy management; detailed subcategorization such as timing or complications was not systematically captured and therefore cannot be further specified). While nurses handled predominantly organizational issues, physicians responded predominantly to medical issues albeit also answering a relevant number of organizational inquiries (42.1%) (Table S3).

**TABLE 1:** Number of care‐related inquiries addressed to MG center.

Total number of care‐related inquiries, *n*	576
Total duration of tasks (hrs)	101.5
Duration tasks per week (hrs)	12.7
Duration tasks per working day^a^ (hrs)	2.5
Duration per task; median [IQR]	5 [5, 10]
Min, max	1, 360

Abbreviations: Hrs, hours; IQR, interquartile range; Max, maximum; Min, minimum.

^a^ Calculated with assumed 5 working days per week.

**TABLE 2:** Topics of care‐related inquiries.

Total number of inquiries recorded, *n*	**576**
Time‐critical inquiries^a^, *n* (%)	**115 (20.0)**
Categories	
Management/Organization, *n* (%)	**319 (55.4)**
Of these time‐critical, *n* (%)	50 (15.7)
Of these related to: (multiple answers possible)	
Appointment, *n* (%)	139 (43.6)
Prescription (drug), *n* (%)	114 (35.7)
Formal requests/application, *n* (%)	34 (10.7)
Prescription (non‐pharmaceutical therapy), *n* (%)	14 (4.4)
Registration for start of new therapies^b^, *n* (%)	16 (5.0)
Other, *n* (%)	15 (4.7)
Medical, *n* (%)	**238 (41.3)**
Of these time‐critical, *n* (%)	61 (25.6)
Of these related to: (multiple answers possible)	
MG worsening, *n* (%)	73 (30.7)
FcRn‐ or complement inhibitors, *n* (%)	46 (19.3)
Treatment plan, *n* (%)	37 (15.5)
Infection, *n* (%)	25 (10.5)
Side‐effects of MG medication, *n* (%)	19 (8.0)
Steroids, *n* (%)	10 (4.2)
NSIT, *n* (%)	10 (4.2)
MG and pregnancy/peri‐partum, *n* (%)	6 (2.5)
MG and comorbidity, *n* (%)	5 (2.1)
Other MG related medical issue, *n* (%)	69 (29.0)
Communication issue, *n* (%)	**6 (1.0)**
Other, *n* (%)	**13 (2.3)**
Role (who took care of/responded to inquiry primarily)	
Physician, *n* (%)	428 (74.3)
Nurse, *n* (%)	148 (25.7)
Communication directly with patient	
Yes, *n* (%)	396 (68.8)
No, *n* (%)	178 (30.9)
With relative/care‐giver, *n* (%)	2 (0.3)
Which communication channel was used by staff to respond? (multiple answers possible)	
Email, *n* (%)	243 (42.2)
Phone, *n* (%)	167 (29.0)
Hospital‐information system, *n* (%)	79 (13.7)
Telemedicine platform (MyaLink), *n* (%)	40 (6.9)
In‐person, *n* (%)	32 (5.6)
Postal, *n* (%)	21 (3.6)
Fax, *n* (%)	3 (0.5)

*Note:* Bold values represent the overall group‐level results, followed by the corresponding subgroup figures.

Abbreviations: FcRn, neonatal fragment crystallizable receptor; MG, myasthenia gravis; NSIT, non‐steroidal immunosuppressive therapy.

^a^ Defined based on clinical judgment as requiring same‐day response (e.g., severe worsening of MG symptoms, issues requiring rapid coordination or adjustment of care like scheduling/modifying complex infusion therapies).

^b^ This relates to a form that has to be filled in before one of the new therapies (e.g., C5‐inhibitors or FcRn‐inhibitors) can be administered at the infusion clinic.

A total of 115 inquiries were classified as time‐critical. Within the subcategory of inquiries related to MG worsening, more than 50% were classified as time‐critical. Email was the most frequently used communication channel, although this was not a standardized communication pathway. Additional colleagues within the MG center were involved in 20.3% of inquiries (e.g., requiring internal coordination or forwarding to the appropriate professional group). (Table S2). Medical inquiries required twice the processing time of organizational inquiries (Table S4).

## 4 Discussion

Our data demonstrate that care‐related communication in a tertiary MG center is heterogeneous, frequently time‐sensitive, and largely conducted through non‐standardized communication channels. One fifth of inquiries required a same‐day response, underscoring the clinical relevance and potential urgency of patient‐initiated contact outside scheduled visits. At the same time, a substantial proportion of inquiries concerned organizational matters, highlighting the administrative workload associated with asynchronous patient communication. Organizational inquiries were more frequently handled by nursing staff, whereas physicians were more often involved in medically complex or prioritization‐related questions. No relevant differences in the communication channels used between professional groups were observed. Similar observations have been reported in studies of patient portal messaging in other medical and neurological conditions where many patient‐initiated messages relate to administrative issues rather than clinical decision‐making [13, 14, 15, 16]. In routine practice, inquiries were typically handled by the MG team member who initially received the message rather than through a structured system. While pragmatic, this decentralized workflow reflects the absence of standardized communication pathways and may contribute to inefficiencies and fragmented coordination of care. The predominance of direct email communication suggests that current pathways are driven by convenience rather than systematic integration into clinical workflows and direct linking to clinical patient data. Therefore, our findings underscore the need for structured communication pathways that ensure timely, appropriate and coordinated responses, rather than stand‐alone symptom‐monitoring tools, particularly as delayed care in MG can lead to serious complications, including myasthenic crises [17]. In this context, several digital and telemedical approaches have been developed to support MG care. These include standardized remote assessments [5, 6], patient‐performed respiratory assessments [7], and app‐ or smartphone‐based tools for symptom documentation [8, 10, 18]. However, these tools primarily address symptom assessment and do not, by themselves, resolve the high proportion of organizational and therapy‐related inquiries. This distinction is important, as many seemingly organizational requests were closely intertwined with clinical decision‐making, for example when coordinating complex infusion therapies.

Consequently, digital solutions for MG care should not be limited to remote symptom monitoring but should integrate structured communication pathways and navigational support. Comprehensive telemedicine platforms tailored to MG, such as MyaLink (RareLink), aim to address this need by the collection of patient‐reported outcome measures (PROMs), wearable‐derived data, and secure patient–physician communication. Additional structured modules for adverse event reporting, infection screening, and therapy‐specific guidance could help standardize information capture beyond symptom severity. Such tools would not replace physician judgment but could support early triage, more efficient routing of requests, and more consistent documentation within specialized MG care. Initial feasibility data support the potential of such integrated approaches [12].

Furthermore, machine‐learning based algorithms using multimodal telemedicine data showed good performance for prediction of MG worsening [19]. This suggests that telemedical platforms in MG may facilitate the early detection of clinical worsening and enable timely interventions, although prospective validation remains necessary.

Wearable technologies such as the OURA ring are being evaluated for longitudinal monitoring of sleep and physical activity in MG patients in a home setting as part of the prospective trial (NCT05992025). The completed study combines a passive monitoring approach with digitally delivered group‐based interventions to assess the feasibility of tracking clinically relevant lifestyle parameters over time. This approach allows for continuous, real‐time data collection, including heart variability, physical activity, and sleep patterns, presenting a promising model for remote disease monitoring. Such technologies may enable more personalized follow‐up and facilitate the early identification of fluctuations in disease activity or objective evidence of fatigue in MG [20].

Recent advances in machine learning have begun to enhance remote assessments by enabling automated detection of facial weakness, ptosis, and dysarthria [9, 21, 22, 23, 24]. These technologies hold promises for early warning systems and longitudinal disease monitoring but will require robust external validation before they can be used in clinical practice. The CAPTURE‐MG study (NCT06743490) uses a cross‐sectional design to assess whether digital features from smartphone can distinguish MG patients from healthy controls, and correlate with disease severity, quality of life, and fatigue alongside a comparison of automated measurements with clinical assessments. However, these technologies require robust external validation before routine clinical use.

These platforms do not address the full repertoire of needs of people with MG, which range from simple administrative tasks such as scheduling or rescheduling a visit to an immediate, urgent need for response to prevent or treat a serious exacerbation or even impending crisis. Further developments should address this hierarchy of workflow options that would make asynchronous care more efficient. It remains to be seen whether artificial intelligence or machine learning models would assist in the development of distinct workflows with the inherent requirement for some overlap and judgment. Furthermore, clinical implementation will depend not only on technical feasibility but also on successful integration into clinical care pathways, supported by healthcare professionals who can guide and contextualize data. Structural barriers include variability in data quality, legal/compliance issues, lack of interoperability between systems, and disparities in patients' digital literacy. Establishing appropriate reimbursement frameworks will also be essential for sustainable, long‐term integration into clinical practice. Our findings therefore suggest that digital tools in MG should not be developed as isolated monitoring applications but as components of coordinated care models that address both symptom monitoring and the substantial administrative and therapeutic communication burden observed in routine practice.

Several limitations should be considered. The study was conducted at a single tertiary center, which limits the generalizability of our findings. This described workflow might reflect the organization of care at this center in Germany and may not be generalizable to other countries or healthcare systems, where administrative responsibilities and clinical workflows can differ. Data collection relied on self‐reporting by clinical staff, which may be subject to reporting bias or incomplete capture during periods of high workload. Furthermore, the observation period was limited to 8 weeks and may not fully reflect long‐term variations in care demands. Another limitation is the lack of patient‐level linkage of communication data. Future studies at the German tertiary center will address this by linking communication data to individual patients using a structured telemedicine platform that captures patient–provider interactions.

In conclusion, care‐related inquiries in MG are diverse, often time‐critical, and handled through a variety of communication channels. Understanding their nature and frequency is essential for designing clinically meaningful and scalable digital health solutions in MG. Such tools must not function in isolation but must be integrated into coordinated care models endorsed and supported by clinical teams. Future studies should address the development and clinical validation of remote outcome measures and integration of digital tools into interdisciplinary workflows.

## Author Contributions

**Martijn R. Tannemaat:** supervision, writing – review and editing. **Anna Rostedt Punga:** writing – review and editing. **Pushpa Narayanaswami:** writing – review and editing. **Lea Gerischer:** conceptualization, investigation, methodology, formal analysis, data curation, writing – review and editing. **Andreas Meisel:** writing – review and editing. **Maike Stein:** writing – original draft, conceptualization, methodology, investigation. **Sophie Lehnerer:** conceptualization, investigation, methodology, writing – review and editing, supervision. **Yvonne J. M. Campman:** writing – review and editing.

## Funding

Argenx provided a grant to support the project but was not involved in the conceptualization/design.

## Conflicts of Interest

M.S. has received speaker's honoraria and honoraria for attendance at advisory boards from Argenx and Alexion. M.S. is shareholder of RareLink digital health GmbH. L.G. has received speaker's honoraria and/or travel and congress fees from Alnylam, Alexion, Roche and UCB and is a shareholder of RareLink digital health GmbH. Y.J.M.C. reports no disclosures relevant to the manuscript. A.R.P. has received speaker's honoraria and honoraria for attendance at advisory boards from Argenx, UCB, Alexion, Novartis and Toleranzia, which are unrelated to this study. P.N. has received grant support from NIH, PCORI and Alexion. P.N. has served as a consultant/advisory board member for Alexion, Amgen, Argenx, Cartesian, CVS Caremark, Dianthus, Immune Abs, Immunovant, Johnson and Johnson, Serono‐EMD, UCB and Viridian. DSMB: Sanofi, Argenx, and NMD pharma. Royalties: Springer Nature.

A.M. received speaker's honoraria, served as an advisor board/DSMB member and consultant, and has received research grants (paid to his institution) from Alexion AstraZeneca Rare Disease, Amgen, argenx, Axunio, Desitin, Genpharm, Grifols, Hormosan, Immunovant, Janssen, Merck, Novartis, Octapharma, Regeneron, Sanofi and UCB. He is chairman of the Association for Research of Myasthenic Syndromes in Germany and member of the medical advisory board of the German Myasthenia Gravis Society. M.R.T. has been paid for consultancies for ArgenX, UCB Pharma, Johnson and Johnson, Peervoice and Medtalks, and has received research funding from Huma, ZonMW, NWO, ArgenX and NMD Pharma. All reimbursements were received by Leiden University Medical Center. He is a member of the European Reference Network for Rare Neuromuscular Diseases [ERN EURO‐NMD]. S.L. received travel/accommodation/meeting expenses from Alexion Astra Zeneca Rare Disease, Argenx, Johnsson&Johnsson, and UCB received speaking honoria and honoria for attendance at advisory boards from Alexion Astra Zeneca Rare Disease, Argenx, Biogen, Hormosan, Huma, Johnsson&Johnsson, Merck, Roche, StreamedUp and UCB. She received financial research support (paid to her institution) from Ad Scientiam, Alexion Pharmaceuticals, Argenx, Hormosan and UCB. S.L. is shareholder of mamahealth GmbH and RareLink digital health GmbH.

## Supporting information


**Table S1:** Evaluation form.
**Table S2:** Involvement of other healthcare professionals and topics of care‐related inquiries.
**Table S3:** Subanalysis which inquiries were handled by physicians or nurses.
**Table S4:** Processing time (duration) per category of care‐related inquires.

## Data Availability

Data not provided in the article may be shared (anonymized) upon reasonable request from the corresponding author.
