# Supplementary material for: Patient Communication Outside of Visits: Implications for Remote Monitoring and Digital Care Models in Myasthenia Gravis
Source: Muscle Nerve. 2026 Jul 16;74(Suppl 1):S118–23. doi: 10.1002/mus.70341 (PMC13599459; doi:10.1002/mus.70341)
Supplement: Supplementary file 1 [file MUS-74-S118-s001.pdf]

Supplementary material

To Clinical Research Article

Patient Communication outside of visits: Implications for remote monitoring and digital care models in Myasthenia Gravis

Authors:

Maike Stein[1,2*], Lea Gerischer[1,2*], Yvonne J.M. Campman[3], Anna Rostedt Punga[5], Pushpa Narayanaswami[6], Andreas Meisel[1,2,7], Martijn R. Tannemaat[3], Sophie Lehnerer[1,2,4]

[1] Charité – Universitätsmedizin Berlin, corporate member of Freie Universität Berlin and Humboldt-Universität zu Berlin, Department of Neurology with Experimental Neurology, Berlin, Germany

[2] Charité – Universitätsmedizin Berlin, corporate member of Freie Universität Berlin and Humboldt-Universität zu Berlin, Neuroscience Clinical Research Center, Berlin, Germany

[3] Department of Neurology, Leiden University Medical Center, Leiden, the Netherlands

[4] Berlin Institute of Health at Charité – Universitätsmedizin Berlin, Digital Health Center, Berlin, Germany

[5] Department of Medical Sciences, Clinical Neurophysiology, Uppsala University, Uppsala, Sweden

[6] Department of Neurology, Beth Israel Deaconess Medical Center/Harvard Medical, School, Boston, Massachusetts, USA

[7] Center for Stroke Research Berlin, Charité – Universitätsmedizin Berlin, Berlin, Germany

*equal contribution

Table S1: Evaluation form.

	Please fill in number and text/explanation where appropriate
**Category:**<br>1 = Management/Organisational;<br>2 = Medical;<br>3 = Communication issue;<br>4 = Other	
Please explain what the inquiry was/what you did e.g. category 1 “prescription (drug)”<br>or category 2 “MG worsening”	
**Time-critical [1]?** (inquiry/task needs to be taken care of on same working day)<br>0 = No; 1 = Yes	
**role of staff member taking care of inquiry**<br>1= Physician; 2 = Nurse	
**Communication directly with the patient?**<br>0 = No; 1 = Yes; 2 = With relative/care-giver	
**How did the communication take place?**<br>1= Phone;<br>2= Email;<br>3= Telemedicine platform (MyaLink);<br>4= Fax;<br>5= Postal;<br>6= Hospital-information system[2];<br>7= In-person;<br>8= Other	
**If** 8= Other please explain	
**Other colleagues/staff involved in task? multiple answers possible**<br>0= No;<br>1= Neurologist;<br>2= Primary care physician;<br>3= Pharmacy;<br>4= Neurological ward;<br>5= Infusion clinic;<br>6= Emergency Room;<br>7= Outpatient Clinic;<br>8= Other hospital;<br>9= Therapist (e.g. physiotherapist, speech therapist);<br>10= Patient organisation;<br>11= Other staff from myasthenia center;<br>12= Other colleagues	
**If** 12= Other colleagues, please explain	
**Topics with other colleagues/staff:**<br>0= No;<br>1= Appointment;<br>2= Medication;<br>3= Diagnostics/results;<br>4= Treatment plan;<br>5= MG worsening;<br>6= Other	
**Minutes** (how long did it take to complete the task?)	

[1] defined based on clinical judgment as requiring same-day response (e.g., severe worsening of MG symptoms, issues requiring rapid coordination or adjustment of care like scheduling/modifying complex infusion therapies)
[2] IT system for hospital data and process management
Abbreviations: MG – Myasthenia gravis

Table S2: Involvement of other healthcare professionals and topics of care-related inquiries

**Other colleagues/staff involved in task?** (multiple answers possible)	
Other staff from myasthenia center, n (%)	117 (20.3)
Infusion Clinic, n (%)	50 (8.7)
Neurologist, n (%)	31 (5.4)
Other colleagues, n (%)	29 (5.0)
Neurological ward, n (%)	24 (4.2)
Outpatient Clinic staff, n (%)	21 (3.6)
Other hospital, n (%)	8 (1.4)
Primary care physician, n (%)	5 (0.9)
Pharmacy, n (%)	5 (0.9)
Emergency Room, n (%)	4 (0.7)
Therapist (e.g. physiotherapist, speech therapist), n (%)	1 (0.2)
**Topics with other colleagues/staff:** (multiple answers possible)	
Appointment, n (%)	104 (18.1)
Treatment plan, n (%)	79 (13.7)
Medication, n (%)	54 (9.4)
MG worsening, n (%)	32 (5.6)
Diagnostics/results, n (%)	22 (3.8)
Other, n (%)	26 (4.5)

Table S3: Subanalysis which inquiries were handled by physicians or nurses

	**Physicians** N= 428	**Nurses** N= 148	**Total** N=576
**Management/Organisation, n (%)**	**180 (42.1)**	**139 (93.9)**	**319 (55.4)**
Of these related to:[a]			
Appointment, n (%)	54 (30.0)	85 (61.2)	139 (43.6)
Prescription (drug), n (%)	74 (41.1)	40 (28.8)	114 (35.7)
Formal requests/application, n (%)	34 (18.9)	0 (0.0)	34 (10.7)
Prescription (non-pharmaceutical therapy), n (%)	1 (0.5)	13 (9.3)	14 (4.4)
Registration for start of new therapies	16 (8.8)	0 (0.0)	16 (5.0)
other, n (%)	14 (7.7)	1	15 (4.7)
**Medical, n (%)**	**235 (54.9)**	**3 (2.0)**	**238 (41.3)**
Of these related to:[a]			
MG worsening, n (%)	72 (30.6)	1 (33.3)	73 (30.7)
FcRn- or complement inhibitors, n (%)	46 (19.6)	0 (0.0)	46 (19.3)
Treatment plan, n (%)	37 (15.7)	0 (0.0)	37 (15.5)
Infection, n (%)	25 (10.6)	0 (0.0)	25 (10.5)
Side-effects of MG medication, n (%)	19 (8.1)	0 (0.0)	19 (8.0)
Steroids, n (%)	10 (4.3)	0 (0.0)	10 (4.2)
NSIT, n (%)	10 (4.3)	0 (0.0)	10 (4.2)
MG and pregnancy/peri-partal, n (%)	6 (2.6)	0 (0.0)	6 (2.5)
MG and comorbidity, n (%)	5 (2.1)	0 (0.0)	5 (2.1)

Other MG related medical issue, n (%)	67 (28.5)	2 (66.7)	69 (29.0)
**Communication issue, n (%)**	**5 (1.2)**	**1 (0.7)**	**6 (1.0)**
**Other, n (%)**	**8 (1.9)**	**5 (3.4)**	**13 (2.3)**

[a] multiple answers possible
Abbreviations: MG – Myasthenia gravis; FcRn - neonatal fragment crystallizable receptor; NSIT - Non-Steroidal Immunosuppressive Therapy

Table S4: Processing time (duration) per category of care-related inquires

**Duration per category (min)**	
**Management/Organisation, median [IQR]**	**5.0 [4.0, 10.0]**
min,max	1.0; 180.0
**Medical, median [IQR]**	**10.0 [5.0, 15.0]**
min,max	2.0; 120.0
**Communication issue, median [IQR]**	**10.0 [4.3; 22.5]**
min,max	2.0; 60.0
**Other, median [IQR]**	**6.5 [5.0; 17.0]**
min,max	5.0; 360.0

Abbreviations: IQR – Interquartile range; min – minimum; max - maximum
